# Optimization of quantitative polymerase chain reactions for detection and quantification of eight periodontal bacterial pathogens

**DOI:** 10.1186/1756-0500-5-664

**Published:** 2012-12-02

**Authors:** Ellen Decat, Jan Cosyn, Hugo De Bruyn, Reza Miremadi, Bart Saerens, Els Van Mechelen, Stefan Vermeulen, Mario Vaneechoutte, Pieter Deschaght

**Affiliations:** 1 Biomedical and Exact Sciences, Faculty of Education, Health&Social Work, University College Ghent, Keramiekstraat 80, Ghent, Belgium; 2 Laboratory Bacteriology Research, Department Clinical Chemistry, Microbiology&Immunology, Faculty of Medicine and Health Sciences, University of Ghent, De Pintelaan 185, Ghent, B-9000, Belgium; 3 Department of Periodontology and Oral Implantology, Dental School, Faculty of Medicine and Health Sciences, University of Ghent, De Pintelaan 185, Ghent, B-9000, Belgium

**Keywords:** QPCR, Periodontal pathogens, Specificity, Quantification limit, Intercycler portability

## Abstract

**Background:**

The aim of this study was to optimize quantitative (real-time) polymerase chain reaction (qPCR) assays for 8 major periodontal pathogens, i.e. *Aggregatibacter actinomycetemcomitans*, *Fusobacterium nucleatum*, *Parvimonas micros*, *Porphyromonas gingivalis*, *Prevotella intermedia*, *Tanerella forsythia* and *Treponema denticola*, and of the caries pathogen *Streptococcus mutans*.

**Results:**

Eighteen different primer pairs were analyzed *in silico* regarding specificity (using BLAST analysis) and the presence of secondary structures at primer binding sites (using mFOLD). The most specific and efficiently binding primer pairs, according to these analyses, were selected for qPCR-analysis to determine amplification efficiency, limit of quantification and intra-run reproducibility. For the selected primer pairs, one for each species, the specificity was confirmed by assessing amplification of DNA extracts from isolates of closely related species. For these primer pairs, the intercycler portability was evaluated on 3 different thermal cyclers (the Applied Biosystems 7300, the Bio-Rad iQ5 and the Roche Light Cycler 480). For all assays on the different cyclers, a good correlation of the standard series was obtained (i.e. r^2^ ≥ 0.98), but quantification limits varied among cyclers. The overall best quantification limit was obtained by using a 2 μl sample in a final volume of 10 μl on the Light Cycler 480.

**Conclusions:**

In conclusion, the proposed assays allow to quantify the bacterial loads of *S. mutans*, 6 periodontal pathogenic species and the genus *Fusobacterium*.This can be of use in assessing periodontal risk, determination of the optimal periodontal therapy and evaluation of this treatment.

## Background

Periodontitis is a multifactorial infectious disease whereby an irreversible destruction of periodontal tissues occurs. This condition is preceded by a reversible state of inflammation of the periodontal tissues, called gingivitis [1]. From a microbiological point of view, this course is characterized by quantitative and qualitative alterations in the microflora of the subgingival environment [2]. The average surface area of the adult human oral cavity has been estimated to amount to approximately 215 cm^2^[3], presenting a vast surface for microbial colonization. A total number of around 700 microbial species has been estimated to populate the numerous surfaces of the oral cavity [4], and major differences can be observed between subjects and even on a site level within one subject [5]. Although most of these bacteria are commensal microorganisms, numerous bacterial species, including several that cannot be grown *in vitro*, have been associated with periodontal health and disease, related to biofilm formation [6-10]. Therefore, assessing the bacterial diversity in the subgingival biofilm may be important for the diagnosis and optimized treatment of periodontal diseases. The total number of microbial cells in subgingival plaque from periodontally healthy subjects has been estimated to amount to 3.3 x 10^9^ cfu/mg, increased to 1.7 x 10^10^ cfu/mg for patients with periodontitis, with considerable inter-subject variation [11]. This increase in microbial counts is also accompanied by a certain shift in the microbial species present [12,13]. Basically, the biofilm continues to develop with increasing biodiversity. So-called periodontal pathogens, mainly including gram negative anaerobic rods and spirochetes (such as *Treponema denticola*) benefit from this phenomenon, especially at the base of the periodontal pocket [13]. Consequently, differences in composition and quantity of the periodontal microflora might be used to explain variations in severity of periodontitis. In spite of the difficulty of cataloguing all the members of the oral microflora and the complexity of their interactions with each other and their human host, certain species have been identified as likely perio-pathogens. For example, there is a strong body of evidence that *Aggregatibacter actinomycetemcomitans*, *Porphyromonas gingivalis, T. denticola* and *Tannerella forsythia* are periodontal pathogens (Slots et al., [14-19]). Whilst *A. actinomycetemcomitans* has been implicated to be responsible for aggressive periodontitis, *P. gingivalis, T. forsythia* and *T. denticola* are more associated with chronic periodontitis [20], although all four species have been implicated in various forms of periodontitis. In addition to these species, moderately strong evidence exists regarding the pathogenicity of certain other bacterial species, such as *Campylobacter rectus*, *Eubacterium nodatum*, *Fusobacterium nucleatum*, *Parvimonas (Micromonas*, *Peptostreptococcus) micros*, *Prevotella intermedia/nigrescens*, *Streptococcus intermedius* and various spirochetes, in some forms of periodontitis [21-29]. Taking these findings into account, the detection and quantification of a limited number of specific bacterial species in subgingival biofilms might be a helpful tool in periodontal risk assessment, determining the optimal periodontal therapy and evaluating the treatment outcome. In this study, we therefore evaluated several qPCR assays for the detection of 8 oral pathogens, i.e. *Aggregatibacter actinomycetemcomitans*, *Fusobacterium* genus, *Parvimonas micros, Porphyromonas gingivalis*, *Prevotella intermedia*, *Streptococcus mutans*, *Tannerella forsythia* and *Treponema denticola*. *S. mutans* was also included given its predominant role in the etiology of dental caries [30]. Periodontitis and caries are the most prevalent oral diseases, still resulting in considerable tooth loss [31].

## Methods

### Bacterial strains

The bacterial strains used in this study for analyzing sensitivity and specificity of the primers are listed in Table 1. Clinical isolates, which were not traceable to the patient, and reference isolates were used. The clinical samples used for the study mentioned that was published elsewhere [32], were covered by the ethical committee approval: B67020097225 (Belgian registration number). These clinical samples were collected from the deepest periodontal pocket per quadrant. A sterile paper point was inserted following supragingival plaque removal and left in situ for about 20 seconds. The paper points were collected in 200 μl of a 20 mM Tris–HCl, pH 8 solution (Merck, Darmstadt, Germany) and stored at −20°C until DNA extraction.

**Table 1:** Bacterial strains and their corresponding collection number used to test sensitivity and specificity of the different primer pairs

**Species**	**Strain**	**Origin**
*Actinobaculum schaalii*	TSW25BA12^a^	human, vagina
*Actinomyces meyeri*	PB2003/218-T1-6^a^	human, vagina
*Actinomyces naeslundii*	CCUG 18310^T^	human, sinus
*Actinomyces neuii*	TSW23BA4^a^	human, vagina
*Actinomyces odontolyticus*	LMG 15953	human, drain after lung resection
*Actinomyces turicensis*	TSW24BA1^a^	human, vagina
*Aggregatibacter actinomycetemcomitans*	DSM 11123	human, subgingival dental plaque
*Agrobacterium radiobacter*	0106 0380^a^	not recorded
*Bacteroides fragilis*	CCUG 4856^T^, 03L2177^a^	human, appendix abscess;
*Bacteroides thetaiotaomicron*	CCUG 34778	human, appendix
*Fusobacterium nucleatum*	CCUG 32989^T^	human, cervico-facial lesion
*Fusobacterium varium*	DSM 19868^T^	human, faeces
*Parvimonas micros*	CCUG 46357^T^	human, purulent pleurisy
*Peptostreptococcus anaerobius*	FWOBV0180^a^	not recorded
*Porphyromonas gingivalis*	CCUG 25893^T^	human, gingival sulcus
*Porphyromonas somerae*	VMF0235S33	human, vagina
*Prevotella melaninogenica*	FWO BV0747^a^	human, vagina
*Prevotella bivia*	FWO BV0913^a^	human, vagina
*Prevotella buccalis*	FWO BV0754^a^	human, vagina
*Prevotella disiens*	VMF 1000SRT31	human, vagina
*Prevotella corporis*	TSW04CA1^a^	human, vagina
*Prevotella intermedia*	CCUG 24041^T^	human, empyema
*Streptococcus agalactiae*	LMG 14694^T^	bovine, milk
*Streptococcus anginosus*	LMG 14502^T^	human, throat
*Streptococcus mitis*	LMG 14557^T^	human, oral cavity
*Streptococcus mutans*	LMG 14558^T^	human, carious dentine
*Streptococcus oralis*	LMG 14532^T^	human, oral cavity
*Streptococcus pneumoniae*	LMG 14545^T^	not recorded
*Streptococcus pyogenes*	LMG 14700^T^	not recorded
*Streptococcus sanguinis*	LMG 14702^T^	human, subacute bacterial endocarditis
*Streptococcus salivarius*	LMG 11489^T^	human, blood
*Streptococcus sobrinus*	LMG14641^T^	human, dental plaque
*Tannerella forsythia*	CCUG 21028A^T^	Human, periodontal pocket
*Treponema denticola*	Oligo^b^	not applicable

Legend

^a^: Patient isolate.; ^b^: *T. denticola* could not be cultured. Therefore, a ds oligonucleotide was used as template for preparing the standard series.

### Extraction of DNA and preparation of standard dilution series

Bacterial genomic DNA used for preparing standard dilution series was extracted with the High Pure PCR Template Preparation Kit (Roche, Basel, Switzerland). Briefly, all strains were grown anaerobically, except for *Streptococcus* spp., which were grown aerobically, on blood agar. Colonies were scraped from plates and suspended in 400 μl PBS. To 200 μl of bacterial suspension, 2 μl mutanolysin (25 U/μl) was added and incubated for 15 min at 37°C. Further DNA extraction was performed according to manufacturers guidelines. The DNA concentration was quantified by spectrophotometric analysis (Nanodrop, Thermo Scientific, Wilmington, DE) and converted from ng/ml to number of genomes/ml by calculating the molecular weight of the genome (ng/genome) and dividing the concentration (ng/ml) by the molecular weight of the genome in order to assign number of genome values to the standard dilution series. Bacterial DNA used for specificity testing was extracted using alkaline lysis. Briefly, strains were grown on agar plates under appropriate conditions, a single colony was picked up and dissolved in 20 μl alkaline lysis buffer (0.25% SDS, 0.05 N NaOH), the mixture was heated for 15 min at 95°C, the tubes were briefly spinned, 180 μl sterile HPLC water was added to neutralize the pH, and the tubes were centrifuged during 5 min at 13000*g* to spin down the bacterial cell debris. The supernatant was used as DNA extract. Tenfold standard dilution series of reference strains were made from genomic DNA extracted from *A. actinomycetemcomitans* DSM 11123, *F. nucleatum* CCUG 32989*, P. micros* CCUG 46357*, P. gingivalis* CCUG 25893, *P. intermedia* CCUG 24041, *S. mutans* LMG 14558^T^ and *T. forsythia* CCUG 21028A^T^*.* Several attempts to grow *T. denticola* from different culture collections failed. Therefore, a tenfold standard dilution series was made of a synthetic ds oligonucleotide. We blasted the primers described by Hyvarinen et al. [33] and found that these were located on the coding domain sequence for a glycosyl transferase, corresponding to region 1470086 – 147094 of strain ATCC 35405 (GenBank: AE017226), which we ordered from Eurogentec (Liège, Belgium). All standard series were diluted in nuclease free water, containing 1 μg/ml calf thymus DNA (Sigma-Aldrich, St. Louis, MO), according to the MIQE guidelines [34]. Calf thymus DNA was added to decrease adherence of the target DNA to the vials, in order to increase reproducibility, especially of the low concentration standards.

### Primers

Primer sequences and amplicons were analysed for specificity using the nucleotide Basic Local Alignment Search Tool and primerBLAST (http://blast.ncbi.nlm.nih.gov/Blast.cgi). The presence of secondary structures was analyzed using mFOLD (http://mfold.rna.albany.edu/?q=mfold).

Table 2 lists the primers that were tested.

**Table 2:** **Primer sequences evaluated for specificity (BLAST) and primer annealing onto secondary structures (mFOLD) by *in silico *analysis for the eight different species**

**Species**	**Primers**	**Target gene**	**Reference**
*Aggregatibacter actinomycetemcomitans*^a^	F: GCGAACGTTACGCGTTTTAC	*waaA*	Hyvarinen et al. [33]
	R: GGCAAATAAACGTGGGTGAC		
*Aggregatibacter actinomycetemcomitans*	F: CTTACCTACTCTTGACATCCGAA	16S rRNA	Maeda et al. [35]
	RV: ATGCAGCACCTGTCTCAAAGC		
*Aggregatibacter actinomycetemcomitans*^b^	F: CAGCATCTGCGATCCCTGTA	*iktA*	Yoshida et al. [36]
	R: TCAGCCCTTTGTCTTTCCTAGGT		
*Fusobacterium* spp.	F: AAGCGCGTCTAGGTGGTTATGT	16S rRNA	Martin et al. [37]
	R: TGTAGTTCCGCTTACCTCTCCAG		
*Fusobacterium* spp.^b^	F: CGCAGAAGGTGAAAGTCCTGTAT	16S rRNA	Suzuki et al. [38]
	R: TGGTCCTCACTGATTCACACAGA		
*Parvimonas micros*	F: AAACGACGATTAATACCACATGAGAC	16S rRNA	Bartz et al. [39]
	R: ACTGCTGCCTCCCGTAGGA		
*Parvimonas micros*^b^	F: AGTGGGATAGCCGTTGGAAA	16S rRNA	Martin et al. [37]
	R: GACGCGAGCCCTTCTTACAC		
*Porphyromonas gingivalis*	F: TGGTTTCATGCAGCTTCTTT	*waaA*	Hyvarinen et al. [33]
	R: TCGGCACCTTCGTAATTCTT		
*Prevotella intermedia*^b^	F: GACCCGAACGCAAAATACAT	*waaA*	Hyvarinen et al. [33]
	R: AGGGCGAAAAGAACGTTAGG		
*Prevotella intermedia*	F: TCCACCGATGAATCTTTGGTC	16S rRNA	Maeda et al. [35]
	R: ATCCAACCTTCCCTCCACTC		
*Tannerella forsythia*^a^	F: CTCGCTCGGTGAGTTTGAA	*waaA*	Hyvarinen et al. [33]
	R: ATGGCGAAAAGAACGTCAAC		
*Tannerella forsythia*	F: GGGTGAGTAACGCGTATGTAACCT	16S rRNA	Shelburne et al. [40]
	R: ACCCATCCGCAACCAATAAA		
*Tannerella forsythia*^b^	F: TCCCAAAGACGCGGATATCA	*bspA* antigen	Morillo et al. [41]
	R: ACGGTCGCGATGTCATTGT		
*Tannerella forsythia*^a^	F: AGCGATGGTAGCAATACCTGTC	16S rRNA	Kuboniwa et al. [42]
	R: TTCGCCGGGTTATCCCTC		
*Tannerella forsythia*^a^	F: ATCCTGGCTCAGGATGAACG	16S rRNA	Suzuki et al. [38]
	R: TACGCATACCCATCCGCAA		
*Treponema denticola*	F: CCTTGAACAAAAACCGGAAA	*waaG*	Hyvarinen et al. [33]
	R: GGGAAAAGCAGGAAGCATAA		
*Streptococcus mutans*^b^	F: AGCCATGCGCAATCAACAGGTT	*gftB*	Yano et al. [43]
	R: CGCAACGCGAACATCTTGATCAG		
*Streptococcus mutans*	F: GCCTACAGCTCAGAGATGCTATTCT	*gftB*	Yoshida et al. [36]
	R: GCCATACACCACTCATGAATTGA		

Legend

^a^: Primer pairs excluded for further *in vitro* testing on the basis of *in silico* analysis.

^b^: Primer pairs excluded for further specificity testing on the basis of amplification efficiency.

### qPCR

Each assay was designed for most efficient amplification with the same thermocycling program: initial dsDNA denaturation (+ activation of hot start enzyme) for 10 min at 95°C, 40 cycles of 15 s at 95°C and 1 min at 60°C, on an ABI 7300 real time PCR system (Applied Biosystems, Carlsbad, CA). The primer concentrations were the same for all assays, *i.e.* 300 nM. Assays were performed in a final volume of 25 μl with a final MgCl_2_ concentration of 3 mM and with 2.5 μl DNA extract, using the SybrGreen qPCR core kit (Eurogentec).

Assays carried out on the LightCycler (LC) 480 thermal cycling system (Roche) were performed in a final reaction volume of 10 μl with 1 or 2 μl of DNA extract (both volumes were tested), using the LightCycler 480 SybrGreen I master mix, with the same primer concentrations and thermocycling program as for the ABI 7300. Assays carried out on the iQ5 thermal cycling system (Bio-Rad Laboratories, Hercules CA) were performed in a final reaction volume of 25 μl with 2.5 μl DNA extract, using the iQ SYBR Green Supermix, with the same primer concentrations and thermocycling program as for the ABI 7300.

## Results

The aim of this study was to optimize quantitative PCR assays (qPCR assays) for 8 important oral bacteria, *i.e. Aggregatibacter actinomycetemcomitans*, *Fusobacterium nucleatum*, *Parvimonas micros*, *Porphyromonas gingivalis*, *Prevotella intermedia*, *Streptococcus mutans*, *Tanerella forsythia* and *Treponema denticola*. *In silico* analysis indicated that it was not possible to develop species specific primers for *F. nucleatum*, based on the 16S rRNA gene. Therefore, *Fusobacterium* genus primers were used, assuming that - when testing oral samples - most signal strength for this qPCR will be caused by the presence of *F. nucleatum*, because this species is the dominant *Fusobacterium* species in oral microflora [44]. Different primer pairs were tested with regard to amplification efficiency, specificity and intercycler portability (robustness), *i.e.* portability between different thermal cyclers.

Initially, the qPCR formats were developed on an ABI 7300 thermal cycling system (Applied Biosystems), on which we first determined the amplification efficiency of the primers. Thereafter, the primer pairs with the best amplification efficiency were used to test intercycler portability by carrying out the PCRs on a LightCycler 480 thermal cycler (Roche) and on an iQ5 thermal cycler (Bio-Rad), with the same cycling parameters as used on the ABI 7300. The thermal cycler that gave the most reproducible and accurate results, was used to test the specificity of the assays.

### Amplification efficiency of different primer pairs

**Figure 1:**
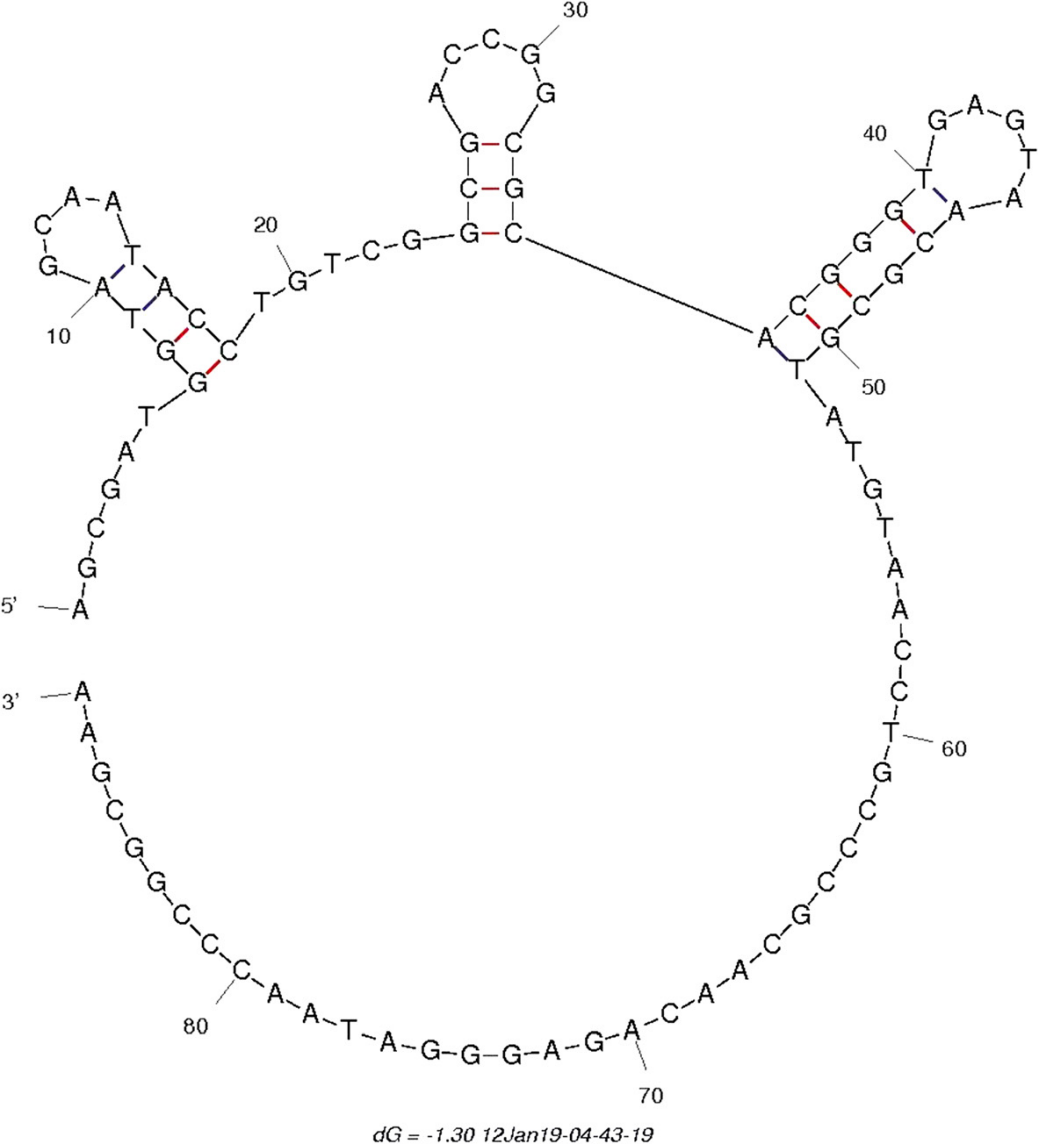
**Analysis by mFold of the secondary structure of the *Tannerella forsythia *16S rRNA gene amplicon, targeted by the primers described by Kuboniwa *et al*. **[42]**.** Folding conditions were adapted to qPCR conditions (see 2.4). Forward primer anneals on bp 1–22 region, which contains a hairpin (bp 7–18).

Bioinformatic analysis (PrimerBLAST, mFold) revealed that, at an annealing temperature of 60°C, some of the primers were annealing on secondary structures in the target genes. An example of annealing on secondary structure is shown in Figure 1 for the *T. forsythia* forward primer that has been proposed by Kuboniwa et al. [42].

As indicated in Table 2, we omitted these primer pairs from subsequent experiments, because annealing of the primers onto secondary structures has been shown to decrease amplification efficiency [45]. First, the amplification efficiency and quantification limit of the selected primer pairs were tested using a 10-fold standard dilution series. The best performing primer pairs were selected on the basis of amplification efficiency, correlation of standard dilution series and quantification limit, the latter defined as the lowest standard dilution that could be included in the standard series without decreasing the amplification efficiency below 95% (Table 3). Moreover, intra-run reproducibility was taken into account (data not presented).

**Table 3:** Primers used for specificity testing, after selection based on amplification efficiency, quantification limit, and intra-run reproducibility (data not presented)

**Species (reference)**	**Correlation standard curve**	**Amplification efficiency (%)**	**Quantification limit (number of bacteria per 25 μl reaction)**
*Aggregatibacter actinomycetemcomitans*[35]	0.99	89	25
*Fusobacterium* spp. [37]	0.99	94	4
*Parvimonas micros*[39]	0.99	91	2
*Porphyromonas gingivalis*[33]	0.99	95	9
*Prevotella intermedia*[35]	0.99	91	11
*Treponema denticola*[33]	0.99	95	150
*Tannerella forsythia*[40]	0.99	93	25
*Streptococcus mutans*[36]	0.98	115	37

### Specificity testing

**Figure 2:**
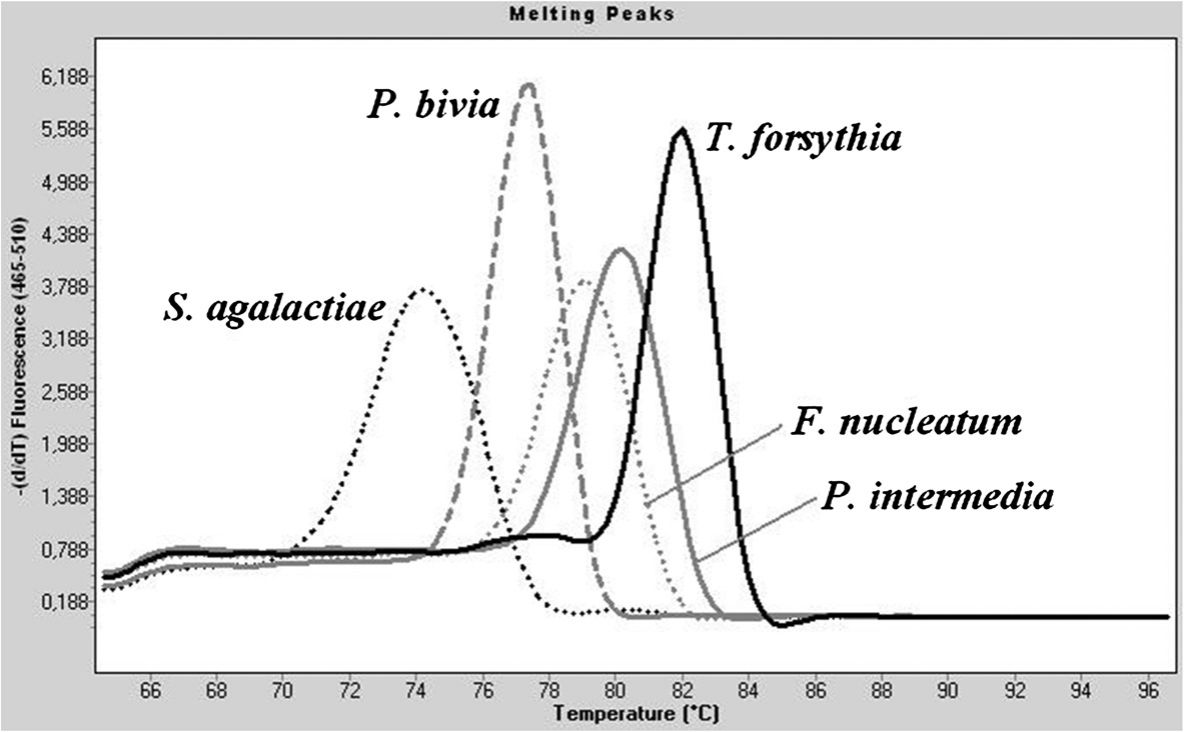
**Melting curve analysis of unspecific amplification products for the *Tannerella forsythia *qPCR **[40]**.** The melting curves presented were drawn by the software of the LC480 cycler after performing the *T. forsythia* qPCR on the species listed in Table 1.

After selection of the primer pairs that enabled amplification of the target species with the same protocol, specificity of the different primer sets was tested by including closely related species (Table 1) in each of the 8 qPCR assays. Assays for *A. actinomycetemcomitans*, *P. micros*, *P. gingivalis* and *P. intermedia* detected only the target species for which they were designed. The assay for the *Fusobacterium* spp. detected also *F. varium*, next to *F. nucleatum*, as expected, since this is a genus specific qPCR. For the assay for *T. forsythia,* some unspecific amplification was observed during the last cycles (35 < C_q_ < 40) for strains of the species *Fusobacterium nucleatum, P. bivia*, *P. intermedia* and *S. agalactiae* (Figure 2). This did not affect the specificity of the *T. forsythia* assay because of the low amplification efficiency. Moreover, the Tm-value of the *T. forsythia* amplicon was situated between 81.96 and 82.02°C, whereas Tm-values for all other species were lower. Every strain included in the specificity testing, except the strains of *P. intermedia* and *A. radiobacter*, gave weak unspecific amplification for the *T. denticola* assay. This could possibly be explained by the formation of primer dimers during the last cycles of the *T. denticola* assay, since the NTC had a high Cq value ( > 40). Still, this little affected the specificity of this assay, first because of the low amplification efficiency for these non- target species (*i.e.*, Cq value below the quantification limit of the assay) and second because the melting profile of the unspecific PCR products was clearly different from that of the target sequence.”

### Intercycler portability (robustness)

After selection of the primer pairs with the highest specificity and amplification efficiency on the ABI 7300 cycler (Table 3), the same assays were carried out on the iQ5 and the LC480 thermal cyclers. In addition, for the LC480, two different DNA extract volumes, i.e. 1 and 2 μl were tested. All qPCR’s on the different cyclers gave good correlation of the standard series (i.e. r^2^ ≥ 0.98), but quantification limits varied between cyclers. The overall best quantification limit was obtained by using a 2 μl sample in a final volume of 10 μl on the LC480 (Table 4).

**Table 4:** Intercycler portability of the different assays on the different thermal cyclers, by comparison of the limits of reliable quantification, i.e. the most diluted standard that could be used to calculate the standard curve, expressed here as number of cells present in the most diluted standard reaction mixture

**Assay**		**ABI 7300 (2.5/22.5)**^**a**^	**iQ5 (2.5/22.5)**	**LC 480 (1/9)**	**LC 480 (2/8)**
**Species**	**Reference**				
*Aggregatibacter actinomycetemcomitans*	Maeda et al. [35]	26	26	10	2
*Fusobacterium* spp.	Martin et al. [37]	4	4	2	3
*Parvimonas micros*	Bartz et al. [39]	2	1	1	2
*Porphyromonas gingivalis*	Hyvarinen et al. [33]	9	90	36	7
*Prevotella intermedia*	Maeda et al. [35]	11	11	4	9
*Streptococcus mutans*	Yoshida et al. [36]	37	37	15	3
*Tannerella forsythia*	Shelburne et al. [40]	25	25	10	2
*Treponema denticola*	Hyvarinen et al. [33]	150	15	6	1

Legend

^a^: Volume of DNA extract (μl)/Volume of total mixture (μl).

## Discussion

Although culture is currently the standard approach for assessing the oral microflora, anaerobic culture, which is required to this aim, is rather costly. Moreover, quantitative culture is very laborious, requiring more culture media, and thus an even more costly technique, with limitations of the number of samples that can be enumerated. Molecular techniques may be valuable alternatives to anaerobic quantitative culture, especially since the availability of quantitative (real-time) PCR (qPCR). Conventional PCR only reveals the presence or absence of a species, while qPCR and DNA-DNA hybridization approaches (Socransky et al. [9,46]) offer (semi-)quantitative data with an acceptable degree of agreement with quantitative culture for most periodontal pathogens [47]. Although a perfect agreement between microbial enumeration techniques seems unlikely [48-50], their availability might become relevant for the clinician, especially when conventional therapeutic modalities have failed. Interestingly, microbial data could also become valuable to predict further periodontal deterioration following active treatment [51].

In order to optimize an assay to detect eight predominant oral pathogens, 8 primer pairs were selected that were run on the same thermocycling program with sufficient amplification efficiency, specificity and sufficient quantification limit. Six of the 8 assays were species specific. For the *T. denticola* and *T. forsythia* assays, some unspecific amplification was observed, but only at C_q_ values of more than 35. This was not an issue, since the last standard included in the standard dilution series, corresponding to one chromosome/reaction for *T. denticola* and 2 chromosomes /reaction *T. forsythia*) had a C_q_ value below 35, such that all fluorescence signals detected after this C_q_ value are considered as not quantifiable. Moreover, melting curve analysis indicated that these unspecific amplification products had melting temperatures that were clearly different from that of the target species.

All assays were evaluated for intercycler portability by running the standard dilution series for each species on three different thermal cyclers, *i.e.* ABI 7300, Bio-Rad iQ5 and LightCycler 480. Highly efficient amplification was obtained on all cyclers, but the LightCycler 480 could detect lower bacterial inocula than the other devices, *i.e.* on average 3.6 chromosomes /reaction, compared to 26 chromosomes/reaction for the iQ5 and 33 chromosomes/reaction for the ABI 7300. In addition, the LightCycler 480 has higher throughput (*i.e.* 384 samples) than the ABI 7300 and Bio-Rad iQ5 devices (*i.e.* 96 samples).

The optimized assays were implemented to evaluate the microbial effects of an essential oils mouth rinse used by patients in supportive periodontal care [32]. Briefly, during a 3-month double-blind randomized placebo-controlled study, these qPCR assays were used to evaluate the microbial effects of an essential oils mouth rinse used as an adjunct approach to mechanical plaque control by patients in supportive periodontal care. Subgingival plaque samples were collected for the quantification of the 8 bacterial species by means of the qPCR formats described here. No significant differences were observed between treatment and placebo groups. Also, there was no significant change over time neither in detection frequency nor load for any of the bacterial species.

## Conclusion

In summary, we present optimized qPCR assays, with high intercycler portability, for direct quantification of 8 bacterial species that have been associated with periodontal disease.

## Competing interests

The authors declare that they have no competing interests.

## Authors’ contributions

Decat E. and Saerens B. carried out the qPCR assays. Decat E. carried out the data analysis and interpretation. Decat E., Vaneechoutte M. and Van Mechelen E wrote the first draft of the manuscript. Decat E., Van Mechelen E. ,Vermeulen S., Cosyn J., Miremadi R., De Bruyn H., Vaneechoutte M. and Deschaght P. conceived the study, participated in its design and coordination and helped to draft the manuscript. All authors read and approved the final manuscript.
